# Capecitabine-Induced Enterocolitis

**DOI:** 10.7759/cureus.22855

**Published:** 2022-03-04

**Authors:** Muzammil Khan, Robert Kleyner, Sadia Abbasi, Asim Haider

**Affiliations:** 1 Internal Medicine, Stony Brook University Hospital, Stony Brook, USA; 2 Internal Medicine, Renaissance School of Medicine at Stony Brook University, Stony Brook, USA; 3 Internal Medicine, BronxCare Health System, New York City, USA

**Keywords:** diarrhea, ileus, chemotherapy-related toxicity, enterocolitis, capecitabine

## Abstract

Capecitabine is an oral fluoropyrimidine carbamate chemotherapy agent approved by the United States Food and Drug Administration (FDA) for the treatment of metastatic colorectal and breast cancer. The common side effects associated with it include gastrointestinal (GI) upset, abdominal pain, palmar-plantar erythrodysesthesia, fatigue, alopecia, leukopenia, neutropenia, thrombocytopenia, anemia, and hyperbilirubinemia. Although GI symptoms are relatively common, enterocolitis is one of the rare side effects of this drug. We present a case of 53-year-old female who developed severe enterocolitis leading to ileus secondary to capecitabine chemotherapy for metastatic breast cancer. She was treated successfully via conservative management.

## Introduction

Capecitabine (Xeloda®, Hoffmann-La Roche) is an oral fluoropyrimidine carbamate chemotherapy agent approved by the United States Food and Drug Administration (FDA) for the treatment of metastatic colorectal and breast cancer [1,2]. The medication was first approved in 1998. In tumor cells, the drug is converted to 5-fluorouracil (5-FU) via a three-enzyme cascade, and subsequently activates p53 by inducing damage to both RNA and DNA; RNA damage results from the incorporation of fluorouridine triphosphate (FUTP) during synthesis and DNA is damaged through both the incorporation of fluorodeoxyuridine triphosphate (FdUTP) into DNA strands, as well as the inhibition of thymidylate synthase [3,4].

Adverse effects reported during clinical trials include gastrointestinal upset, abdominal pain, palmar-plantar erythrodysesthesia, fatigue, alopecia, leukopenia, neutropenia, thrombocytopenia, anemia, and hyperbilirubinemia [5]. However, cases of capecitabine-induced enterocolitis, pneumatosis intestinalis, pancreatitis, small bowel obstruction, and large bowel perforation have been reported in the literature [6-11]. In this case study, we present a case of capecitabine-induced ileus/enteritis in a 53-year-old female undergoing treatment for metastatic breast cancer.

## Case presentation

The patient is a 53-year-old female with metastatic breast cancer who presented to the emergency department with a six-day history of spasmodic abdominal pain, which worsened acutely. The pain was localized to the mid-abdomen and was worsened by food and fluid intake. The patient also endorsed nausea, vomiting, and diarrhea; the latter was controlled with loperamide taken as needed. A subsequent review of systems was negative for fever, chest pain, dyspnea, hematemesis, bilious vomiting, hematochezia, dysuria, and hematuria. The patient denied any other symptoms.

The patient was receiving capecitabine (Xeloda®) monotherapy for metastatic breast cancer to the spine. Prior breast cancer treatments included bilateral mastectomies at age 45-46, chemotherapy, radiotherapy, and hormone therapy. The patient also received palbociclib (IBRANCE®) therapy and was enrolled in a clinical trial. Other medications included pantoprazole and ondansetron.

**Figure 1:**
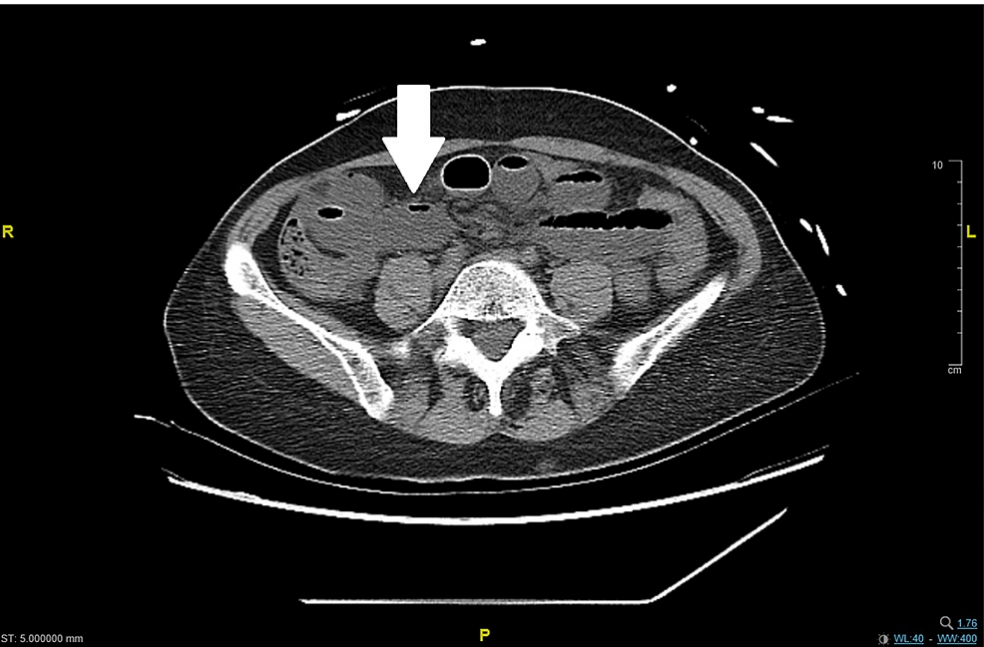
Computed tomography (CT) scan of the abdomen showing dilated intestinal loops (white arrow)

On presentation, vital signs were notable for tachycardia (heart rate of 135 beats per minute), as was a blood pressure of 102/67 mmHg. Electrolyte were within normal range (potassium: millimol/L, sodium: 140 milliequivalents per liter, calcium: 9.0 millimol/L). The physical exam was notable for mild, diffuse abdominal tenderness on palpation, but was otherwise unremarkable. The patient was resuscitated with intravenous (IV) administration 2 L of lactated ringers and was scheduled for a computed tomography (CT) scan of the chest, abdomen, and pelvis without contrast, given that the patient reported a contrast allergy. The CT scan was significant for mildly dilated and fluid-filled small bowel loops with air-fluid levels concerning ileus and enteritis. The abdominal CT scan also revealed mild ascites, as well as three liver lesions with low attenuation (Figure 1). The CT chest without contrast was otherwise unremarkable.

The patient was admitted to the general medicine service and received dicyclomine and pantoprazole, in addition to supportive treatment with bowel rest, IV hydration, and electrolyte replenishment. Pain was managed with IV acetaminophen and nausea was managed with IV ondansetron. Treatment with capecitabine was also discontinued. The patient was able to tolerate a liquid and solid diet on the second and third days of hospitalization, respectively. Following a three-day hospital stay, the patient was discharged on dicyclomine, pantoprazole, ondansetron, and acetaminophen, and was advised to follow-up with her primary oncology team.

## Discussion

Adverse gastrointestinal (GI) effects associated with capecitabine administration are common. In patients undergoing treatment for metastatic breast cancer with capecitabine monotherapy, nausea and diarrhea were reported in more than half of patients (57% and 53%, respectively). Other commonly reported side effects include vomiting (37%), stomatitis (24%), abdominal pain (20%), constipation (15%), and dyspepsia (8%). Capecitabine monotherapy, when used for any indication, was associated with ileus in 0.3% of patients. Gastroenteritis, ascites, and gastric ulcer each occurred in 0.1% of treated patients [5]. However, given the relatively high frequency of abdominal pain, it may be possible that cases of ileus and gastroenteritis may not have been recognized and were, therefore, underreported.

Several cases of capecitabine-associated intestinal pathologies have been reported in literature. A case presentation and narrative literature review published in January 2022 characterizes 21 reported cases of capecitabine-associated enterocolitis. Of these cases, only four (19%) occurred when capecitabine was used as first-line, second-line, or neoadjuvant therapy. Most patients presented with watery diarrhea, abdominal pain, and distension, although dehydration, fever, nausea, vomiting, and hematochezia have also been reported. Patients were usually managed with IV hydration, total parenteral nutrition, and broad-spectrum antibiotics. In patients who did not respond to initial therapy, treatment with oral budesonide, octreotide, and cholestyramine was attempted, but tended not to show additional benefit [6]. Previous literature reviews examining patients with terminal ileitis have also demonstrated a possible female predominance, with nine out of 12 of reviewed capecitabine-induced ileitis cases occurring in females [12].

These findings were generally reflected in our patient, with several exceptions. Our patient presented with diarrhea, vomiting, and signs of dehydration, but did not present with a fever. Hence, no antibiotics were administered, given that there was unlikely to be an infectious etiology. In addition to supportive treatments, our patient was also administered dicyclomine, an antispasmodic medication used to manage irritable bowel syndrome [13,14]. Although this regimen was well-tolerated, further evidence is needed prior to recommending dicyclomine as therapy for patients with capecitabine-associated intestinal pathologies.

The underlying mechanism behind capecitabine-induced intestinal toxicity is complex. Previous studies have hypothesized that it may be a result of the mitotic arrest of crypt cells, decreased aquaporin expression due to increased gene expression of inflammatory cytokines such as Interleukin-4 (IL-4), the mitotic arrest of intestinal smooth muscle cells, and direct endothelial toxicity secondary to the generation of reactive oxygen species [6,12]. 5-FU, the drug’s active metabolite, has also been reported to result in coronary artery vasospasm and may therefore lead to ischemia due to endothelial damage of intestinal vasculature [6,15]. Furthermore, mutations in DPYD, which codes for dihydropyrimidine dehydrogenase, have been previously associated with severe adverse reactions to capecitabine, including ileitis and cytomegalovirus-associated enterocolitis [16,17]. Hence, several medical associations recommend that all patients undergoing treatment with 5-FU drugs and prodrugs be tested for DPYD mutations [18].

## Conclusions

Enterocolitis is likely an underreported side effect of capecitabine therapy and should be investigated as a more common adverse reaction. We argue that clinicians should set a lower threshold to obtain an imaging workup in patients treated with capecitabine therapy, especially in patients who present with spasmatic abdominal pain, nausea, and vomiting. Although most patients can be managed with conservative therapy, dicyclomine should also be investigated as a potential treatment of capecitabine-associated abdominal pain. Furthermore, a meta-analysis of available case studies would be helpful in further characterizing the clinical presentation and treatment of capecitabine-associated GI pathologies.
